# Characterization of Two Dehydrogenases from *Gluconobacter oxydans* Involved in the Transformation of Patulin to Ascladiol

**DOI:** 10.3390/toxins14070423

**Published:** 2022-06-21

**Authors:** Edicon T. S. Chan, Yan Zhu, Xiu-Zhen Li, Ting Zhou, Stephen Y. K. Seah

**Affiliations:** 1 Department of Molecular and Cellular Biology, University of Guelph, Guelph, ON N1G 2W1, Canada; edicontz@uoguelph.ca; 2 Guelph Research and Development Centre, Agriculture and Agriculture-Food Canada, Guelph, ON N1G 5C9, Canada; yan.zhu@agr.gc.ca (Y.Z.); xiu-zhen.li@agr.gc.ca (X.-Z.L.)

**Keywords:** mycotoxin, patulin, ascladiol, *Gluconobacter oxydans*, detoxification, enzyme, dehydrogenase

## Abstract

Patulin is a mycotoxin that primarily contaminate apples and apple products. Whole cell or cell-free extracts of *Gluconobacter oxydans* ATCC 621 were able to transform patulin to E-ascladiol. Proteins from cell-free extracts were separated by anion exchange chromatography and fractions with patulin transformation activity were subjected to peptide mass fingerprinting, enabling the identification of two NADPH dependent short chain dehydrogenases, GOX0525 and GOX1899, with the requisite activity. The genes encoding these enzymes were expressed in *E. coli* and purified. Kinetic parameters for patulin reduction, as well as pH profiles and thermostability were established to provide further insight on the potential application of these enzymes for patulin detoxification.

## 1. Introduction

Patulin (4-hydroxy-4H-furo [3,2-c]pyran-2(6H)-one) is a mycotoxin produced by several genera of fungi, such as *Penicillium*, *Aspergillus*, *Paecilomyces*, and *Byssochlamys* [1,2,3,4]. The electrophilic α,β-unsaturated lactone in patulin is reactive, contributing to its ability to induce double strand DNA breaks, modify sulfhydryl compounds, and cause cellular oxidative stress [5]. Numerous foods can be contaminated by patulin including fruit juices, jams, grain products, and cheese [6,7,8,9,10,11] but apples and apple-based products are of particular concern due to their prevalence in the human diet, particularly in infants and children [10,12,13,14,15,16]. When ingested orally, ulcers, inflammation, and hemorrhages within the intestine are some of the most significant symptoms of toxicity [17]. In North America and the EU, a 50 μg/kg patulin limit in apple juice has therefore been established by regulatory agencies [18,19,20].

Various physical and chemical based methods have been employed in attempts to reduce patulin contamination in apple products. However, there are concerns about the safety and nutritional content of the food products after treatment [21,22]. Biological transformation of patulin, either by whole cells or enzymes, are alternative methods of patulin detoxification that has garnered significant interest in recent years.

**Figure 1:**
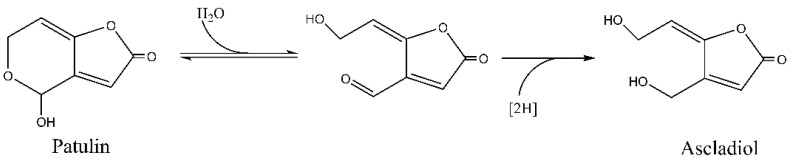
Transformation of patulin to E-ascladiol. The reaction involves opening of the hemiacetal ring followed by reduction of the aldehyde to an alcohol.

Recently, there have been several enzymes involved in patulin removal that have been identified or isolated. These include NrdA from *Enterobacter clocae* subsp. *dissolvens* [23], PcCRG1 from *Pichia carribica* [24], an orotate phosphoribosyltransferase from *Rhodotorula mucilaginosa* [25], and porcine pancreatic lipase [26]. Certain yeasts, such as *Saccharomyces cerevisiae* [27], *Kodameae ohmeri* [28], *Candida guilliermondii* [29], and *Sporobolomyces* specifically accumulate the compound, ascladiol, when incubated with patulin [30]. This is thought to occur by the opening of the patulin hemiacetal ring, followed by the reduction of the aldehyde group to an alcohol (Figure 1) [30]. The E-isomer of ascladiol is produced from patulin but it can be converted to the Z-isomer through catalysis by cellular sulfhydryl compounds such as glutathione and cysteine [31]. Both E-ascladiol and Z-ascladiol were shown to be non-cytotoxic in human cell lines [32]. To date, only the short chain reductase/dehydrogenase enzyme from *Candida guilliermondii* has been experimentally verified to catalyze the reduction of patulin to ascladiol [33]. However, the specific activity and kinetic parameters for this enzyme towards patulin has not been reported.

*Gluconobacter oxydans* is a rare example of a bacterial species that can convert patulin to ascladiol [34]. The Gram negative bacteria have been found in rotten apples that contained significant levels of patulin [34]. Since *Gluconobacter* species can be found in fermented food and are generally considered to be safe, *G. oxydans* has been explored as a possible biocontrol agent against patulin and the patulin producing fungi, *Penicillium expansum* [35].

Here we describe the successful identification of several dehydrogenases responsible for transforming patulin to ascladiol in *G. oxydans* 621 strain. Two enzymes from the short-chain dehydrogenase/reductase (SDR) family, GOX0525 and GOX1899, were overproduced in recombinant *E. coli* and purified, enabling their kinetic and biochemical characterization.

## 2. Results

### 2.1. Analysis of Patulin Transformation by G. oxydans 621

**Figure 2:**
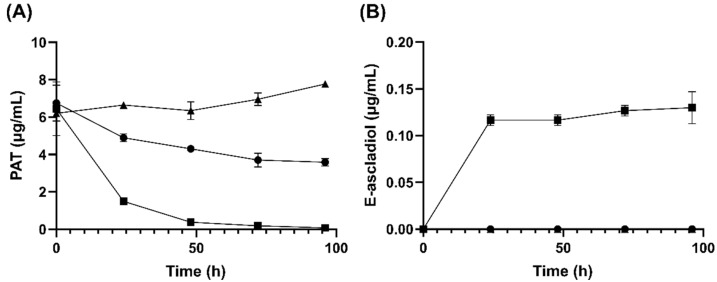
Kinetics of (**A**) patulin (PAT) concentration change in media and (**B**) E-ascladiol production by *G. oxydans* strain 621. Live or dead *G. oxydans* ATCC 621 culture were incubated with 10 µg/mL patulin at 30 °C. Five hundred µL samples were taken every 24 h and extracted with 500 µL ethyl acetate. The organic phase was dried and then resuspended in 50 µL 10% acetonitrile solution before analysis by HPLC using a C18 column. Data were from ● no cells control, ■ live *G. oxydans*, ▲ autoclaved, dead *G. oxydans*.

Cell suspensions of *G. oxydans* 621 were able to remove more than 90% of a 10 μg/mL concentration of patulin within 48 h and 100% within 96 h as determined from HPLC analysis. Concomitant to the disappearance of patulin is the formation of E-ascladiol in the mixture (Figure 2). A reduction in patulin concentration from 10 μg/mL to 8 μg/mL in assays containing dead cells was observed initially that is likely due to adsorption as no ascladiol formation was detected [36]. The adsorbed patulin appeared to be released at a slow rate over the time course of the experiment. Overall the results suggest that E-ascladiol formation from patulin is not spontaneous and is likely due to an enzyme catalyzed reaction.

**Figure 3:**
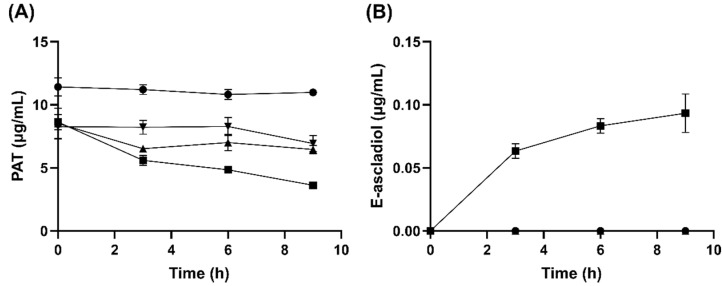
Effects of NADH or NADPH on patulin transformation activity of *G. oxydans* strain 621 cell lysates. (**A**) Patulin (PAT) transformation activity (**B**) E-ascladiol formation. Reaction contains 500 µM NADH or NADPH, 10 µg/mL patulin, and 40 µL/mL cell lysate in 20 mM HEPES buffer, pH 6.5. Samples were taken every 3 h from a 0 h to 9 h time interval and were extracted with ethyl acetate. The organic phase was dried and then resuspended in 50 µL 10% acetonitrile solution before analysis by HPLC using a C18 column. Only lysates incubated with NADPH produced E-ascladiol. Data were from ● no lysate control, ■ lysate with NADPH, ▲ lysate with NADH and ▼ lysate, with no cofactors.

Cell-free lysate of *G. oxydans* ATCC 621 with NADPH was able to reduce 10 µg/mL of patulin in solution by more than 50% within 9 h (Figure 3A). In comparison, there was no significant difference (*p* > 0.05) in patulin transformation whether or not NADH cofactor was present (Figure 3A). Furthermore, E-ascladiol formation only occurred in the presence of NADPH cofactor (Figure 3B). This suggests that the enzyme(s) responsible for patulin transformation to E-ascladiol localized in the cytoplasm of *G. oxydans* and require(s) NADPH as a cofactor.

**Figure 4:**
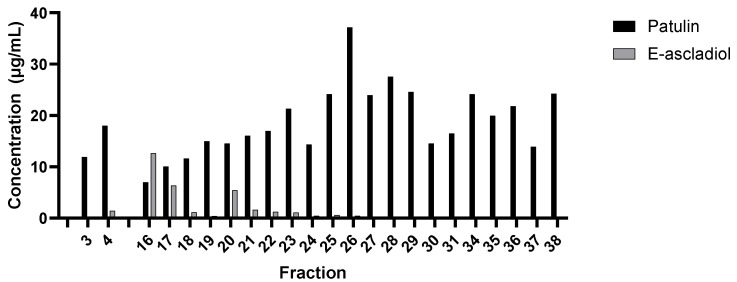
Analysis of ascladiol formation by proteins separated by anion exchange chromatography. One hundred µL from each fraction obtained from the anion exchange chromatography was added to 20 µg/mL patulin and 500 µM NADPH in 20 mM HEPES buffer, pH 6.5 for a total reaction volume of 500 µL. Reaction was incubated at 30 °C for 24 h and the amounts of patulin and E-ascladiol (indicated in µg/mL) were determined by HPLC using patulin and E-ascladiol with known concentrations as standards.

Proteins from *G*. *oxydans* cell lysate were separated by anion exchange chromatography using a gradient of sodium chloride concentration. Fractions were then analyzed for their ability to catalyze ascladiol formation from patulin in the presence of NADPH. Fractions 16, 17, and 20, formed 12.6 μg/mL, 6.35 μg/mL, and 5.43 μg/mL of ascladiol, respectively, from an initial 20 μg/mL of patulin in 24 h (Figure 4). Proteins in fractions 16 and 17 were eluted from the anion exchange column at approximately 0.03 M NaCl and the proteins in fraction 20 were eluted at approximately 0.14 M NaCl.

### 2.2. Protein Identification

Fraction 20 and the combined fractions 16 and 17 were subjected to tryptic peptide-tandem mass spectrometry analysis followed by peptide matching to the protein sequences of *G. oxydans* 621H [37]. Twenty-one proteins were detected in fractions 16/17, four of which are putative oxidoreductases (Supplementary Table S1, marked by asterisk). One protein (AAW60890.1) shares 45% sequence similarity to human succinic semialdehyde dehydrogenase [38] that oxidizes an aldehyde substrate to a carboxylic acid instead of the reduction of aldehyde to an alcohol required for the transformation of patulin to E-ascladiol. Another oxidoreductase (AAW60280.1) shares homology to FMN dependent enzymes and is not NADPH dependent. Only AAW61452.1 and AAW60303.1 share homology to nicotinamide cofactor dependent oxidoreductases that can reduce carbonyl oxygens to alcohols.

Most dehydrogenases utilize a Rossman-fold consisting of 6–7 β-strands flanked by 3–4 α-helices from each side to bind to the nicotinamide cofactor. The 2′ and 3′ hydroxyls of the adenine ribose of NAD(H) usually hydrogen bond with an acidic aspartate or glutamate residue after the second β-strand [39]. This negatively charged acidic residue will repel negatively charged 2′-phosphate of NADP(H). Thus, NAD(P)H specific enzymes normally have a replacement of this acidic residue with a neutral residue. An additional positively charged residue in the vicinity is usually present that forms a salt bridge with the negatively charged 2′ phosphate [39]. Based on these criteria, sequence alignment of AAW61452.1 and AAW60303.1 with their homologs, lactaldehyde dehydrogenase and β-keto acyl carrier protein, respectively, showed that only AAW60303.1 (also annotated as GOX0525) is NADP(H) dependent (Supplementary Table S1).

For fraction 20, there were 67 proteins identified from peptide fingerprinting. Among the 10 oxidoreductases in the list (Supplementary Table S2, marked by asterisk) are a nitroreductase (AAW60608.1), a flavoenzyme (AAW59748.1), and a hydroxyacid reductase that do not catalyze the reduction of an aldehyde to an alcohol. Based on sequence alignments, two of the remaining 7 oxidoreductases (AAW60096.1 and AAW60639.1) are Rossman fold containing enzymes and are predicted to be NAD(H) dependent. Therefore, the predicted NADP(H) dependent dehydrogenases/reductase selected as possible candidates for patulin transformation were GOX0314 (AAW60097.1), GOX0716 (AAW60493.1), GOX1462 (AAW61211.1), GOX1615 (AAW61356.1), and GOX1899 (AAW61637.1).

### 2.3. Analysis of Ascladiol Formation by Whole Recombinant E. coli Cells

Genes encoding putative patulin transforming enzymes (GOX0525, GOX0314, GOX0716, GOX1462, GOX1615, GOX1899), as determined through peptide fingerprinting of protein fractions from anion exchange chromatography, were amplified from genomic DNA of *G. oxydans* by PCR and ligated into the *E. coli* expression plasmid pET28a.

**Figure 5:**
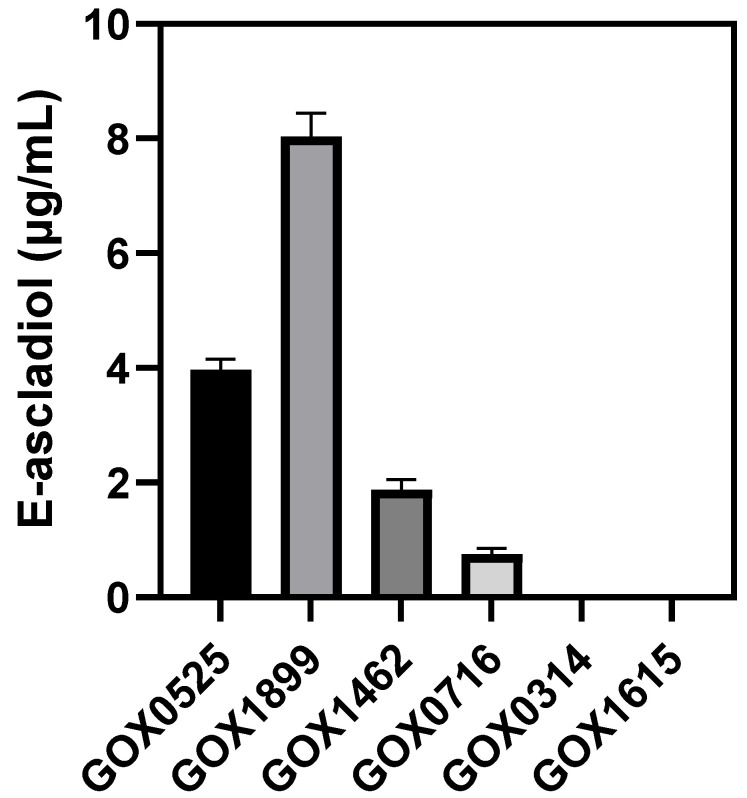
Determination of E-ascladiol formation from patulin by recombinant *E. coli* LOBSTR-BL21 (DE3) expressing various *G. oxydans* enzymes. Recombinant *E. coli* LOBSTR-BL21 (DE3) cells were resuspended at a concentration of 50 mg/mL in 20 mM MES buffer, pH 5. Resuspended cell pellet buffer was incubated with 10 µg/mL patulin, and 500 µM NADPH for 24 h. Samples were analyzed by HPLC in triplicate. Labels on the x-axis indicate the *G. oxydans* protein produced in each recombinant *E. coli*.

To efficiently screen which genes encode enzymes that can transform patulin, whole recombinant *E. coli* cells containing the respective *G. oxydans* genes in plasmids were evaluated for E-ascladiol formation. The only *E. coli* strains capable of producing E-ascladiol were those containing GOX0525, GOX1899, GOX1462 and GOX0716 (Figure 5). No detectable E-ascladiol was observed for *E. coli* strains producing GOX0314 and GOX1615.

### 2.4. Purification of Recombinant Enzymes That Transform Patulin to E-Ascladiol

**Figure 6:**
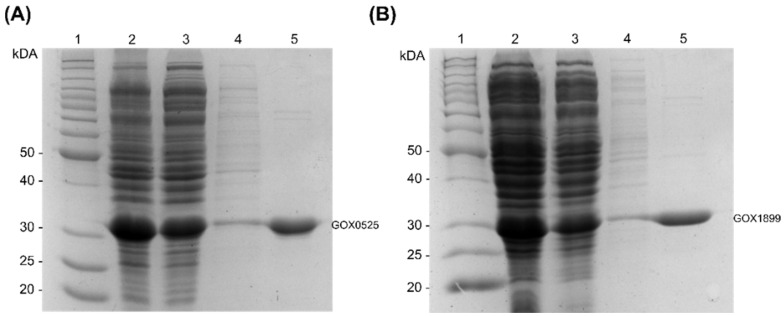
SDS-PAGE analysis of fractions from the purification of (**A**) GOX0525 and (**B**) GOX1899. Lanes (1) Benchmark™ protein ladder (Invitrogen) serving as a molecular marker (2) cell-free extract, (3) flow-through, (4) 20 mM imidazole wash, (5) 150 mM imidazole elution. The band corresponding to the expected size of GOX0525 and GOX1899 are labelled accordingly.

Attempts to purify GOX1462 from recombinant *E. coli* resulted in protein aggregation while GOX0716 was previously reported to be expressed in an insoluble form in *E. coli* [40]. Therefore, we focused on GOX0525 and GOX1899 that produced the two highest amounts of ascladiol from the whole cell assays. The two N-terminal His-tagged enzymes were successfully purified from recombinant *E. coli* by Ni-NTA chromatography. SDS-PAGE analysis showed that GOX0525 and GOX1899 have an apparent molecular mass close to their predicted molecular mass of 29 kDa (Figure 6).

**Figure 7:**
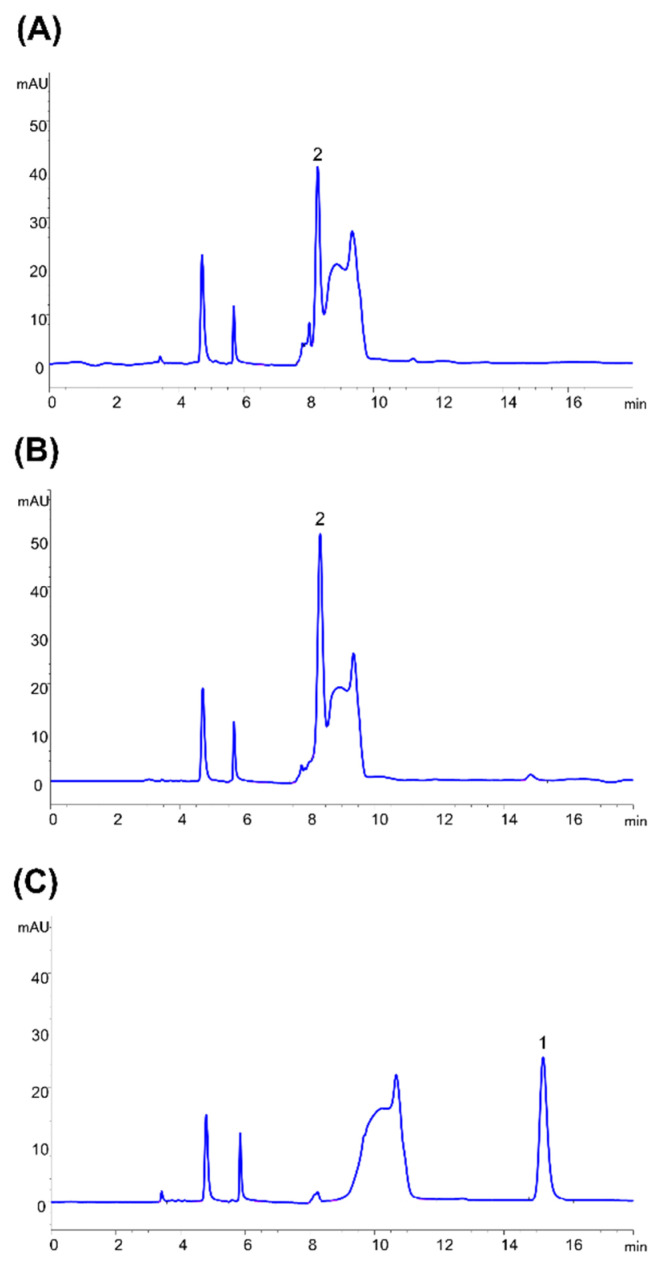
HPLC chromatogram of assay mixture containing 500 μM NADPH and 10 μg/mL patulin in a 20 mM MES buffer, pH 5 with either (**A**) 10.3 μg GOX0525 (**B**) 47 μg GOX1899 or (**C**) no enzyme added. Reaction mixtures were incubated for 24 h at 30 °C before HPLC analysis. Patulin (1) and E-ascladiol (2) were eluted at 14.5 and 8-min retention times, respectively.

The purified enzymes reduced 10 µg/mL patulin to ascladiol in the presence of 500 µM NADPH as determined by HPLC (Figure 7). Reaction mixture was also subjected to LC-MS revealing the presence of a compound with an *m/z* of 157.048 in the positive ion mode, corresponding to ascladiol in the protonated form. This species was absent in the no enzyme control. Instead, the major peak from the no enzyme control has an *m/z* of 155.03 in the positive ion mode, corresponding to patulin in the protonated form.

### 2.5. Kinetic Analysis

The kinetic parameters for patulin transformation were determined for both GOX0525 and GOX1899 using a pH 6 buffer. Data for GOX0525 were fitted into a Michaelis–Menten equation, giving an apparent *K_M_* of 8.64 ± 0.89 mM and an apparent *k*_cat_ of 10.1 ± 0.7 s^−1^. The catalytic efficiency (*k_cat_*/*K_M_*) of GOX0525 against patulin is 1.17 × 10^3^ s^−1^ M^−1^. Data of GOX1899 were also fitted to the Michaelis–Menten equation, giving an apparent *K_M_* value of 7.26 ± 0.54 mM and an apparent *k*_cat_ of 0.425 ± 0.016 s^−1^. The catalytic efficiency of GOX1899 against patulin is 5.85 × 10 M^−^^1^ s^−^^1^, which is approximately 20 times lower than the catalytic efficiency of GOX0525.

### 2.6. pH Dependence of GOX0525 and GOX1899 Activity

**Figure 8:**
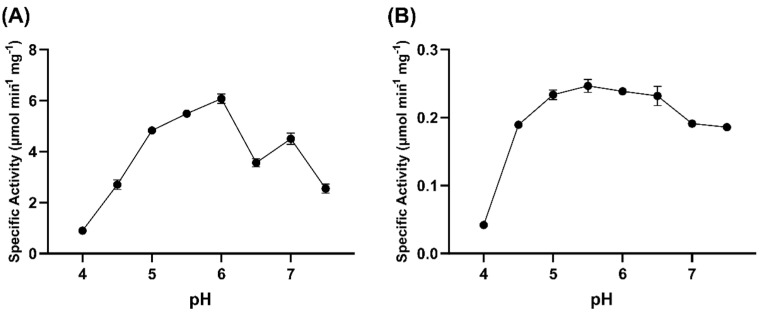
The pH dependence of patulin transformation activity of (**A**) GOX0525 and (**B**) GOX1899. Assay consisted of 250 µM NADPH and 100 µg patulin in a tricomponent buffer containing 0.1 M Tris, 0.05 MES, and 0.05 M acetic acid. The error bars indicate standard deviation for each point performed in triplicate.

GOX0525 has optimal activity at pH 6, with an apparent specific activity of 6.08 ± 0.18 µmol min^−1^ mg^−1^ (Figure 8A). GOX1899 has optimal activity at pH 5.5, with an apparent specific activity of (2.47 ± 0.01) × 10^−1^ µmol min^−1^ mg^−1^ (Figure 8B) but it maintained relatively high activity over a broad pH range from pH 5 to 6.5.

### 2.7. Thermostability

**Figure 9:**
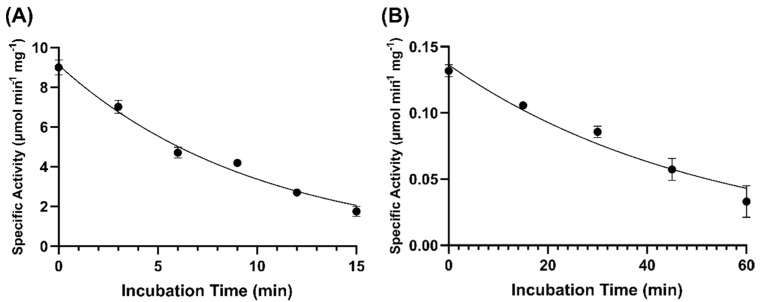
Time course of activity of (**A**) GOX0525 incubated at 55 °C, (**B**) GOX1899 incubated at 80 °C. Aliquots were removed at each time point, and the specific activity of the enzyme was determined spectrophotometrically at 25 °C. Assay consisted of 250 µM NADPH and 200 µg patulin in 0.1 M Tris, 0.05 MES, and 0.05 M acetic acid pH 6.0 buffer. The error bars indicate standard deviation for each point performed in triplicate.

GOX0525 was incubated at 55 °C at various time intervals and tested for activity at 25 °C. Data acquired were plotted and fitted to an exponential decay equation, giving a half-life of 6.97 min (Figure 9A). In comparison, when aliquots of GOX1899 were incubated at 55 °C over a period of 60 min, a reduction in activity of only approximately 25% was detected after 1 h. GOX1899 was also incubated at 80 °C at various time intervals giving a half-life of 36.1 min (Figure 9B), indicating that GOX1899 is more thermostable than GOX0525.

## 3. Discussion

*Gluconobacter oxydans* is an obligate aerobic bacterium found naturally in environments such as soil, fruit, juice, beer, and wine [41,42]. *G. oxydans*, along with other acetic acid bacteria, are unique in that they do not completely oxidize carbon sources, such as carbohydrates and alcohols, under optimum growth conditions [43]. It is therefore unsurprising that *G. oxydans* possess a number of dehydrogenases/reductases, four of which were shown in this study to be capable of transforming patulin to E-ascladiol. One of these enzymes is GOX0525, a dehydrogenase homologous to 3-ketoacyl-[acyl carrier protein] reductase, FabG. FabG has been found to have broad specificity, with the ability to utilize fatty acid substrates of varying lengths that are covalently bound, via thioester linkages, to acyl carrier proteins (ACP) [44]. The 3-keto acyl-ACP substrates are reduced to 3-hydroxy ACP during fatty acid synthesis in prokaryotes. The plasticity of FabG active site to accommodate substrates of varying sizes contributes to its promiscuous activity towards various carbonyl substrates. In fact, GOX0525 has been previously found to stereospecifically reduce a range of ketones, such as ethyl 2-oxo-4-phenylbutyrate (*k_cat_*/*K_M_*: 1.719 × 10^4^ M^−1^ s^−1^), ethyl 4-chloroacetoacetate (*k_cat_*/*K_M_*: 4.09 × 10^3^ M^−1^ s^−1^), and 4-phenyl-2-butanone (*k_cat_*/*K_M_*: 300 M^−1^ s^−1^) to their corresponding alcohols [40]. Aldehydes, such as benzaldehyde, acetaldehyde, and pentanal, were, however, not substrates of GOX0525 [45]. The reduction of patulin by GOX0525 reported in this paper therefore represents the first instance of an aldehyde reduction reaction catalyzed by GOX0525.

The other dehydrogenase identified, GOX1899, is a NADPH-dependent aldehyde reductase and has also been characterized previously against 16 different aldehydes, with a preference for medium/long-chain aldehydes such as heptanal (*k_cat_*/*K_M_*: 1.5 × 10^4^ M^−1^ s^−1^) [46]. Schweiger and Deppenmeier (2010) speculate that GOX1899 may be responsible for removing toxic aldehydes from incomplete oxidation of metabolites in *G. oxydans* [46].

GOX0525 and GOX1899 both belong to the short-chain dehydrogenase/reductase (SDR) family of enzymes. SDRs carry out NAD(H) or NADP(H)-dependent oxidation/reduction reactions and typically form dimers or tetramers [47]. The family is diverse with some members sharing only 15–30% sequence identity [47,48]. Despite the sequence divergence among many SDRs, the tertiary structures of the enzymes are highly conserved [48]. Most SDRs have a conserved GxxxGxG motif near the N-terminal [47,49], allowing for a compact βαβ fold [50,51]. The motif is also part of the Rossmann fold, which is ubiquitous in enzymes that bind nicotinamide cofactors [50,51,52]. The residue after the second β-strand of the Rossman fold confer cofactor specificity [39] enabling our identification of NADPH dependent enzymes from the list of dehydrogenases isolated after anion exchange chromatography separation of *G. oxydans* cell lysate.

The only other enzyme that has been experimentally verified to transform patulin to ascladiol is another NADPH dependent short-chain dehydrogenase/reductase, named CgSDR, isolated from *Candida guilliermondii* [33]. The specific activity and kinetic parameters for CgSDR was not determined and therefore it is not possible to compare the catalytic efficiencies of CgSDR with GOX0525 and GOX1899. However, 150 µg/mL CgSDR was reported to transform a third of a 50 μg/mL of patulin mixture in 24 h [33]. Under similar conditions, lower concentrations of the *Gluconobacter* enzymes (10.3 µg/mL GOX0525 and 47 µg/mL GOX1899) were able to transform patulin completely to ascladiol within 24 h, suggesting that the *Gluconobacter* enzymes are more efficient for patulin detoxification.

While GOX0525 and GOX1899 are promising candidates for transformation of patulin, application of these enzymes to detoxify patulin in an apple juice medium requires consideration of other factors. The instability of the expensive NADPH cofactor in acidic conditions found in apple juice is a significant problem. Wu et al. (1986) determined that for every pH unit decrease from pH 7.5, there is a five-fold increase in the degradation rate constant of NADPH [53]. Protein engineering techniques could be applied in the future to alter the coenzyme preference of the enzymes from NADPH to the less expensive and more acid stable, NADH. Alternatively, the enzymes can potentially be applied during the washing step of apple juice processing. Sydenham et al. (1995) demonstrated a significant mean reduction in patulin on apples after washing, from 920 ppb to 190 ppb [54]. The authors also note that this patulin decrease corresponded to the accumulation of patulin in the circulating flume water used for washing [54]. The dehydrogenases from *G. oxydans* described here may therefore be potentially useful for reducing patulin load in flume water that has a neutral pH.

## 4. Materials and Methods

### 4.1. Bacterial Strains

*Gluconobacter oxydans* ATCC 621 was purchased from Cederlane (Burlington, ON, Canada). Competent *E. coli* DH5α and *E. coli* LOBSTR-BL21 (DE3) cells were purchased from Thermo-Fisher Scientific and Kerafast Inc. (Boston, MA, USA), respectively.

### 4.2. Chemicals

Patulin was purchased from Cayman Chemical (Ann Arbor, MI, USA). FastDigest restriction enzymes and buffer were purchased from Thermo Scientific (Ottawa, ON, Canada). T4 DNA ligase, Q5 High-Fidelity DNA polymerase, and Q5 High GC Enhancer were purchased from New England Biolabs (Pickering, ON, Canada). Ni-NTA Superflow resin was purchased from Qiagen (Mississauga, ON, Canada). All other chemicals were obtained from Fisher Scientific (Toronto, ON, Canada) or Sigma-Aldrich (Oakville, ON, Canada), unless otherwise stated.

### 4.3. High-Performance Liquid Chromatography and Mass Spectrometry

Samples and standards were filtered through a 0.45 µm syringe filter (PVDF ACRODISC LC 13, Whatman, Florham Park, NJ, USA) and were analyzed using an HPLC system (Agilent Technology 1200 Series, Palo Alto, CA, USA) equipped with a quaternary pump, an inline degasser, and a diode array detector. A Phenomenex^®^ 4 µm Jupiter Proteo 90 Å (250 × 4.6 mm) with a C18 guard column (Torrance, CA, USA) was used for the separation. The compound of interest, patulin and E-ascladiol, were eluted using binary mobile phase set at a flow rate of 1.0 mL/min, and detected at 276 and 270 nm, respectively. The mobile phase was acetonitrile: water (10:90 by volume) and the injection volume was 10 μL.

Liquid chromatography–mass spectrometry analyses were performed on an Agilent 1200 HPLC liquid chromatograph interfaced with an Agilent UHD 6530 Q-Tof mass spectrometer at the Mass Spectrometry Facility of the Advanced Analysis Centre, University of Guelph. A C18 column (Agilent Poroshell 120, 50 mm × 3 mm 2.7 µm) was used for chromatographic separation with the following solvents water with 0.1% formic acid for A and acetonitrile with 0.1 formic acid for B. The mobile phase gradient was as follows: initial conditions, 5% B increasing to 95% B in 15 min followed by column wash at 95% B and 10-min re-equilibration. The flow rate was maintained at 0.4 mL/min. The mass spectrometer electrospray capillary voltage was maintained at 4.0 kV and the drying gas temperature at 325 °C with a flow rate of 12 L/min. Nebulizer pressure was 40 psi and the fragmentor was set to 160. Nitrogen was used as nebulizing, drying gas, and collision-induced dissociation gas. The mass-to-charge ratio was scanned across the *m/z* range of 50–2000 *m/z* in 4 GHz (extended dynamic range positive-ion auto MS/MS mode. Two precursor ions per cycle were selected for MS^2^ fragmentation scanning from 25 to 2000 *m/z*. The instrument was externally calibrated with the ESI TuneMix (Agilent). The sample injection volume was 5 µL. The data were analyzed using the Agilent Qualitative Analysis software 10.

### 4.4. Screening for Whole G. oxydans Cells for Patulin Transformation Activity

Patulin was added to 10 mL of an overnight culture of *G. oxydans* ATCC 621 grown in YPM broth to a final concentration of 10 µg/mL patulin and the mixture was incubated at 30 °C with shaking. Ten milliliter of autoclaved bacterial culture (121 °C, 15 psi, 30 min) was used as a negative control. At specific time points (0 h, 24 h, 48 h, 72 h, 96 h), 500 µL of the mixture was removed and added to 500 µL of ethyl acetate. The organic fractions were transferred to a new tube and allowed to evaporate in the fume hood overnight. The dried samples were then dissolved in 50 µL 10% ACN solution before HPLC analysis. Each sample was done in triplicates.

### 4.5. Evaluating Patulin Transformation Activity in G. oxydans Cell Lysates

*G. oxydans* cell pellet from a 100 mL culture was resuspended in 20 mM HEPES pH 6.5 buffer to a final cell density of 100 mg/mL. Five milliliter of 0.1 mm glass beads (BioSpec, Bartlesville, OK, USA) was added to the resuspended pellet and homogenized six times in 30 s intervals using a Mini-BeadBeater 16 (BioSpec, Bartlesville, OK, USA). The mixture was then centrifuged at 9605× *g* for 3 min and the supernatant filtered through a 0.22 µm filter (Fisher-Scientific) to obtain cell free lysate for in vitro patulin transformation activity.

To assess cofactor requirements for patulin transformation, reactions contained 500 µM NADH or NADPH, 10 µg/mL patulin, and 40 µL/mL cell lysate in 20 mM HEPES pH 6.5 buffer. At various time points (0 h, 3 h, 6 h, 9 h), 500 μL of the mixture was added to 500 µL ethyl acetate and the samples processed similarly to the procedure described for the analysis of whole cell transformation.

### 4.6. Anion Exchange Chromatography and Analysis of Fractions for Patulin Transformation Activity

Chromatography was performed on an ÅKTA Explorer 100 apparatus (Amersham Pharmacia Biotech, Baie d’Urfé, QC, Canada). Buffers containing 20 mM sodium HEPES (pH 7.5) were used throughout the purification procedure. The *G. oxydans* cell pellet (approximately 4 g wet weight) was resuspended in buffer and disrupted by being passed through a French press three times at an operating pressure of 15,000 psi. The cell debris was removed by centrifugation at 17,500× *g* for 10 min. The clear supernatant was filtered through a 0.45 µm filter and was loaded onto a Source 15Q (Amersham Pharmacia Biotech) anion exchange column (2 cm × 13 cm; 40 mL resin), which had been equilibrated with Buffer A (20 mM sodium HEPES, pH 7.5). The column was washed with 2 column volumes of Buffer A, followed by a linear gradient from 0% Buffer B (20 mM sodium HEPES, 1 M NaCl, pH 7.5) to 60% over 5 column volumes. Fractions of 10 mL volumes were collected.

One hundred µL from each fraction collected from the anion exchange chromatography was added to 20 µg/mL patulin, and 500 µM NADPH in 20 mM HEPES pH 6.5 buffer for a total reaction volume of 500 µL. All tubes were placed in a shaker and incubated at 30 °C for 24 h. Afterwhich, 500 µL of acidified acetonitrile (ACN + 1% acetic acid), was added to quench each reaction and samples were then analyzed by HPLC.

### 4.7. Identification of Proteins in Anion Exchange Chromatography Fractions by Mass-Spectrometry

Proteins were denatured in 6 M urea/2 M thiourea (in 10 mM HEPES, pH 8.0), reduced in 10 mM dithiothreitol (in a 50 mM ammonium bicarbonate buffer), and alkylated in 55 mM iodoacetamide (in 50 mM ammonium bicarbonate buffer before digestion with lysine C for 3 h, followed by an overnight digestion with trypsin (Princeton Separations Adelphia, NJ, USA). Trifluoroacetic acid was added to stop digestion, and solutions were dried under vacuum.

Mass spectrometry identification of peptides were performed similarly to the procedure described for the analysis of patulin and ascladiol by LC-MS described previously except that the mobile phase gradient was as follows: initial conditions, 2% B, increasing to 45% B in 40 min and then to 55% B in next 10 min, followed by column wash at 95% B and 10-min re-equilibration. The first 2 and last 5 min of gradient were sent to waste and not the spectrometer. The flow rate was maintained at 0.2 mL/min. The mass spectrometer electrospray capillary voltage was maintained at 4.0 kV and the drying gas temperature at 350 °C with a flow rate of 13 L/min. Nebulizer pressure was 40 psi and the fragmentor was set to 150. The mass-to-charge ratio was scanned across the *m/z* range of 300–2000 *m/z* in 4 GHz (extended dynamic range positive-ion auto MS/MS mode). Three precursor ions per cycle were selected for fragmentation.

Raw data files were loaded directly into the PEAKS Xpro software (Bioinformatics Solutions Inc., Waterloo, ON, Canada) where the data were refined and subjected to de novo sequencing and matching with *G. oxydans* 621H protein sequences. The following modifications were considered within the search parameters: methionine oxidation, deamidation of asparagine, glutamine and carbamidomethylation of cysteine residues. The tolerance values used were 15 ppm for parent ions and 0.05 Da for fragment ions.

### 4.8. Gene Cloning

Genes corresponding to the proteins of interest identified from peptide fingerprinting were amplified by PCR using EcoRI digested *G. oxydans* ATCC 621 genomic DNA and 0.4 mM dNTP, 1 nmol/mL forward and reverse primers (Table 1), 5X Q5 reaction buffer, 5X Q5 High GC Enhancer, and 1 U of Q5 High-Fidelity DNA Polymerase in a final volume of 50 µL. A touchdown PCR protocol was used consisting of 95 °C for 3 min, followed by 30 cycles of denaturation at 94 °C for 30 s, annealing for 30 s, and extension for 1 min at 72 °C. Annealing temperature was lowered by 0.3 °C per cycle from an initial temperature of 65 °C. After which 10 cycles of denaturation at 94 °C for 30 s, annealing at 52 °C for 30 s, and extension for 1 min at 68 °C was performed, followed by a final extension at 68 °C for 10 min. The amplified PCR fragments were digested with restriction enzymes and then inserted into pET28a.

### 4.9. Recombinant E. coli Patulin Transformation Assay

Recombinant *E. coli* cells were resuspended at a concentration of 50 mg/mL in 20 mM MES pH 5.0 buffer. The suspension was incubated with 10 µg/mL patulin, and 500 µM NADPH in a shaker at 30 °C and samples were taken at 0 h and 24 h time points. Acidified acetonitrile (ACN + 1% acetic acid), was added in a 1:1 ratio to quench each reaction in the sample collected. Patulin and ascladiol form the mixture was analysed by HPLC.

### 4.10. Expression and Purification of Recombinant G. oxydans Genes in E. coli LOBSTR-BL21 (DE3)

Ten mL each of overnight starter culture of recombinant *E. coli* LOBSTR-BL21 (DE3) was used to inoculate 4 × 1 L of LB broth. The culture was incubated in a 37 °C shaker until a OD_600_ of 0.6 was reached. Protein expression was induced with 1 mM isopropyl-β-D-1-thiogalactopyranoside (IPTG), and the culture was further incubated at 15 °C for 20 h.

Cell pellets were re-suspended in 20 mM HEPES pH 8.5 buffer and lysed by French press at 15,000 psi. The crude cell lysate was centrifuged twice at 39,191× *g* for 10 min to remove the insoluble fractions. Buffer used in the purification protocol contains 50 mM sodium phosphate and 300 mM sodium chloride, pH 8.0. The cell-free extract was passed through a 0.45 µm syringe filter (Fisher Scientific) and incubated for 1 h at 4 °C with Ni^2+^-NTA resin and buffer containing 20 mM imidazole. The mixture was poured into a gravity column and washed with several column volumes of the same buffer. His-tagged proteins were eluted using a buffer containing 150 mM imidazole.

The eluted protein was exchanged into 20 mM sodium HEPES pH 7.5 buffer by dilution into a stirred cell with a YM10 filter (Amicon, Miami, FL, USA). GOX0525 was buffer exchanged into 20 mM HEPES pH 7.2 containing 120 mM potassium chloride, 1 mM EDTA, and 1 mg/mL bovine serum albumin (BSA) in accordance to the procedure described for the purification of a homolog of the enzyme [55].

### 4.11. Assessment of Enzyme Purity and Concentration

Samples of protein were mixed with 2× SDS-PAGE loading buffer and boiled for 2 min. Boiled samples were loaded onto a 10% SDS-PAGE gel along with a Benchmark™ protein ladder (Invitrogen, Waltham, MA, USA) serving as a molecular marker. Electrophoresis was performed 10 mA for 2 h. Gels were stained using Coomassie blue for 30 min, and destained with 20% methanol, 10% acetic acid. Destained gels were incubated overnight in 10% glycerol and imaged the next day using the Gel Doc system (Bio-Rad Inc.). Protein purity was assessed with the Image Lab software (Bio-Rad Inc., Hercules, CA, USA). Protein concentration was determined with the Bradford assay using bovine serum albumin as protein concentration standards [56].

### 4.12. Enzyme Assays

In vitro assays to verify patulin transformation into ascladiol were performed in glass vials. Reaction mixture contained either 10.3 μg GOX0525 or 47 μg GOX1899 suspended in 20 mM MES buffer, pH 5, containing 500 μM NADPH and 10 μg/mL patulin. Reaction was incubated at 30 °C for 24 h. Following this, the mixture was filtered though a YM-10 filter before analysis by LC-MS.

Standard kinetic enzyme assays were performed in triplicate at 25 °C using a Varian Cary 3 spectrophotometer with a thermojacketed cuvette holder using a tricomponent buffer (0.1 M Tris, 0.05 M 2-(*N*-morpholino)ethanesulfonic acid (MES), 0.05 M acetic acid, pH 6.0) and 250 µM NADPH. The enzyme activity was measured by monitoring NADPH oxidation at 340 nm with an extinction coefficient of 6220 M^−1^ cm^−1^. Kinetic parameters were determined using varying concentrations of patulin and data were fitted to a Michaelis–Menten equation by non-linear regression using the GraphPad Prism. No NADPH oxidation occurred in the presence of patulin under assay conditions prior to addition of the enzyme as determined spectrophotometrically.

The pH dependence of enzymes were determined using a three component buffer (0.1 M Tris, 0.05 M 2-(*N*-morpholino)ethanesulfonic acid (MES), 0.05 M acetic acid) ranging from pH 4.0 to 7.5 and 100 µg/mL patulin.

Thermostability was carried out by incubating aliquots of enzymes at 55 °C or 80 °C. At different time intervals, the respective enzyme aliquot was removed and cooled on ice for 1 min prior to enzyme activity measurement using the standard assay with 200 µg/mL patulin.

## Figures and Tables

**Table 1:** Sequences of primers used in this study.

Gene Name	Primer	Sequence
GOX0525	Forward	GGC G**GA ATT C**AT GAC CCA CAG AGT AGC GCT C
	Reverse	GGG C**AA GCT T**CA GGC GAC GAG ACC GCC
GOX1899	Forward	GCG G**CA TAT G**AC CAC GGA CAA CAT CG’
	Reverse	GGG C**AA GCT T**AA AAC TCC TGT CTG GTC GG
GOX1462	Forward	CGC C**CA TAT G**CA GTA TCG TCA GCT TGG
	Reverse	GGG C**AA GCT T**CA TTT TTT CGT TTC AAG GGC CTC
GOX0716	Forward	CGC T**CA TAT G**GC CGA TCA CAG CAT C
	Reverse	CGC C**AA GCT T**AC TTC GTC GTG TAC CCT CC
GOX0314	Forward	CCG C**CA TAT G**TT CGC CAT GCA GCT C
	Reverse	CCG C**AA GCT T**CA CGG CAG CAA TAC GGC
GOX1615	Forward	GGG A**GA ATT C**AG TCC CGT GCC GG
	Reverse	CGC T**CT CGA G**TC AGT CCC GTG CCG GG

Bold sequences indicate introduced restriction sites (NdeI, EcoRI, XhoI, or HindIII) to facilitate insertion of PCR amplified fragments into *E. coli* expression plasmid vector, pET28a.

## Data Availability

Data were contained within the article and the Supplementary Materials.

## Supplementary Materials

The following supporting information can be downloaded at: www.mdpi.com/article/10.3390/toxins14070423/s1, Table S1: Mass Spectrometry Peptide Fingerprinting Results for Fraction 16/17; Table S2: Mass Spectrometry Peptide Fingerprinting Results for Fraction 20. References [57,58,59,60,61] are cited in the Supplementary Materials.

## References

[1] Anderson M.S., Dutton M.F., Harding K. (1979). Production and Degradation of Patulin by *Paecilomyces* Species, a Common Contaminant of Silage. J. Sci. Food Agric..

[2] Lovett J., Thompson R.G. (1978). Patulin Producution by Species of *Aspergillus* and *Penicillium* at 1.7, 7.2 and 12.8 C. J. Food Prot..

[3] Rice S.L., Beuchat L.R., Worthington R.E. (1977). Patulin Production by *Byssochlamys* Spp. in Fruit Juices. J. Food Sci..

[4] Roland J.O., Beuchat L.R. (1984). Biomass and Patulin Production by *Byssochlamys nivea* in Apple Juice as Affected by Sorbate, Benzoate, SO_2_ and Temperature. J. Food Sci..

[5] Fliege R., Metzler M. (2000). Electrophilic Properties of Patulin. Adduct Structures and Reaction Pathways with 4-Bromothiophenol and Other Model Nucleophiles. Chem. Res. Toxicol..

[6] Bullerman L.B., Olivigni F.J. (1974). Mycotoxin Producing-Potential of Molds Isolated From Cheddar Cheese. J. Food Sci..

[7] Harwig J., Blanchfield B.J., Scott P.M. (1978). Patulin Production by *Penicillium roqueforti* Thom from Grape. Can. Inst. Food Sci. Technol. J..

[8] Reiss J. (1976). Prevention of the Formation of Mycotoxins in Whole Wheat Bread by Citric Acid and Lactic Acid (Mycotoxins in Foodstuffs. IX). Experientia.

[9] Rychlik M., Schieberle P. (1999). Quantification of the Mycotoxin Patulin by a Stable Isotope Dilution Assay. J. Agric. Food Chem..

[10] Scott P.M., Miles W.F., Toft P., Dubé J.G. (1972). Occurrence of Patulin in Apple Juice. J. Agric. Food Chem..

[11] Zouaoui N., Sbaii N., Bacha H., Abid-Essefi S. (2015). Occurrence of Patulin in Various Fruit Juice Marketed in Tunisia. Food Control.

[12] de Sylos C.M., Rodriguez-Amaya D.B. (1999). Incidence of Patulin in Fruits and Fruit Juices Marketed in Campinas, Brazil. Food Addit. Contam..

[13] Gökmen V., Acar J. (1998). Incidence of Patulin in Apple Juice Concentrates Produced in Turkey. J. Chromatogr. A.

[14] Leggott N.L., Shephard G.S. (2001). Patulin in South African Commercial Apple Products. Food Control.

[15] Ritieni A. (2003). Patulin in Italian Commercial Apple Products. J. Agric. Food Chem..

[16] Tangni E.K., Theys R., Mignolet E., Maudoux M., Michelet J.Y., Larondelle Y. (2003). Patulin in Domestic and Imported Apple-Based Drinks in Belgium: Occurrence and Exposure Assessment. Food Addit. Contam..

[17] Puel O., Galtier P., Oswald I.P. (2010). Biosynthesis and Toxicological Effects of Patulin. Toxins.

[18] European Commission Commission Regulation (EC) (2006). No. 1881/2006 of 19 December 2006 Setting Maximum Levels for Certain Contaminants in Foodstuffs. Off. J. Eur. Union.

[19] FDA CPG Sec.510.150 Apple Juice, Apple Juice Concentrates and Apple Juice Products—Adulteration with Patulin. https://www.fda.gov/regulatory-information/search-fda-guidance-documents/cpg-sec510150-apple-juice-apple-juice-concentrates-and-apple-juice-products-adulteration-patulin.

[20] Health Canada List of Contaminants and Other Adulterating Substances in Foods. https://www.canada.ca/en/health-canada/services/food-nutrition/food-safety/chemical-contaminants/contaminants-adulterating-substances-foods.html.

[21] Zhong L., Carere J., Lu Z., Lu F., Zhou T. (2018). Patulin in Apples and Apple-Based Food Products: The Burdens and the Mitigation Strategies. Toxins.

[22] Ioi J.D., Zhou T., Tsao R., Marcone M.F. (2017). Mitigation of Patulin in Fresh and Processed Foods and Beverages. Toxins.

[23] Xing M., Li B., Chen Y., Tian S. (2020). Ribonucleoside Diphosphate Reductase Plays an Important Role in Patulin Degradation by *Enterobacter cloacae* Subsp. dissolvens. J. Agric. Food Chem..

[24] Wang K., Zheng X., Yang Q., Zhang H., Apaliya M.T., Dhanasekaran S., Zhang X., Zhao L., Li J., Jiang Z. (2019). S-Adenosylmethionine-Dependent Methyltransferase Helps *Pichia caribbica* Degrade Patulin. J. Agric. Food Chem..

[25] Tang H., Li X., Zhang F., Meng X., Liu B. (2019). Biodegradation of the Mycotoxin Patulin in Apple Juice by Orotate Phosphoribosyltransferase from *Rhodotorula mucilaginosa*. Food Control.

[26] Liu B., Peng X., Meng X. (2018). Effective Biodegradation of Mycotoxin Patulin by Porcine Pancreatic Lipase. Front. Microbiol..

[27] Moss M.O., Long M.T. (2002). Fate of Patulin in the Presence of the Yeast *Saccharomyces cerevisiae*. Food Addit. Contam..

[28] Dong X., Jiang W., Li C., Ma N., Xu Y., Meng X. (2015). Patulin Biodegradation by Marine Yeast *Kodameae ohmeri*. Food Addit. Contam.—Part A.

[29] Chen Y., Peng H.-M., Wang X., Li B.-Q., Long M.-Y., Tian S. (2017). Biodegradation Mechanisms of Patulin in *Candida guilliermondii*: An ITRAQ-Based Proteomic Analysis. Toxins.

[30] Ianiri G., Idnurm A., Wright S.A.I., Durán-Patrón R., Mannina L., Ferracane R., Ritieni A., Castoria R. (2013). Searching for Genes Responsible for Patulin Degradation in a Biocontrol Yeast Provides Insight into the Basis for Resistance to This. Appl. Environ. Microbiol..

[31] Sekiguchi J., Shimamoto T., Yamada Y., Gaucher G.M. (1983). Patulin Biosynthesis: Enzymatic and Nonezymatic Transformations of the Mycotoxin (E)-Ascladiol. Appl. Environ. Microbiol..

[32] Tannous J., Snini S.P., el Khoury R., Canlet C., Pinton P., Lippi Y., Alassane-Kpembi I., Gauthier T., el Khoury A., Atoui A. (2017). Patulin Transformation Products and Last Intermediates in Its Biosynthetic Pathway, E- and Z-Ascladiol, Are Not Toxic to Human Cells. Arch. Toxicol..

[33] Xing M., Chen Y., Li B., Tian S. (2021). Characterization of a Short-Chain Dehydrogenase/Reductase and Its Function in Patulin Biodegradation in Apple Juice. Food Chem..

[34] Ricelli A., Baruzzi F., Solfrizzo M., Morea M., Fanizzi F.P. (2007). Biotransformation of Patulin by *Gluconobacter oxydans*. Appl. Environ. Microbiol..

[35] Bevardi M., Frece J., Mesarek D., Bošnir J., Mrvčić J., Delaš F., Markov K. (2013). Antifungal and Antipatulin Activity of *Gluconobacter oxydans* Isolated from Apple Surface. Arh. Hig. Za Rada I Toksikol..

[36] Wang L., Wang Z., Yuan Y., Cai R., Niu C., Yue T. (2015). Identification of Key Factors Involved in the Biosorption of Patulin by Inactivated Lactic Acid Bacteria (LAB) Cells. PLoS ONE.

[37] Prust C., Hoffmeister M., Liesegang H., Wiezer A., Fricke W.F., Ehrenreich A., Gottschalk G., Deppenmeier U. (2005). Complete Genome Sequence of the Acetic Acid Bacterium *Gluconobacter oxydans*. Nat. Biotechnol..

[38] Kim Y.-G., Lee S., Kwon O.-S., Park S.-Y., Lee S.-J., Park B.-J., Kim K.-J. (2009). Redox-Switch Modulation of Human SSADH by Dynamic Catalytic Loop. EMBO J..

[39] Carugo O., Argos P. (1997). NADP-Dependent Enzymes. I: Conserved Stereochemistry of Cofactor Binding. Proteins.

[40] Chen R., Liu X., Lin J., Wei D. (2014). A Genomic Search Approach to Identify Carbonyl Reductases in *Gluconobacter oxydans* for Enantioselective Reduction of Ketones. Biosci. Biotechnol. Biochem..

[41] Macauley S., Mcneil B., Harvey L.M. (2001). The Genus *Gluconobacter* and Its Applications in Biotechnology. Crit. Rev. Biotechnol..

[42] de Ley J., Swings J., Whitman W.B. (1984). Family VI, Actinobacteriacaea. Genus II, Gluconobacter. Bergey’s Manual of Systematic Bacteriology.

[43] Deppenmeier U., Hoffmeister M., Prust C. (2002). Biochemistry and Biotechnological Applications of *Gluconobacter* Strains. Appl. Microbiol. Biotechnol..

[44] Shanbhag A.P. (2019). FabG: From a Core to Circumstantial Catalyst. Biotechnol. Lett..

[45] Chen R., Liu X., Wang J., Lin J., Wei D. (2015). Cloning, Expression, and Characterization of an Anti-Prelog Stereospecific Carbonyl Reductase from *Gluconobacter oxydans* DSM2343. Enzym. Microb. Technol..

[46] Schweiger P., Deppenmeier U. (2010). Analysis of Aldehyde Reductases from *Gluconobacter oxydans* 621H. Appl. Microbiol. Biotechnol..

[47] Jörnvall H., Persson B., Krook M., Atrian S., Roser Gonzàilez-Duarte R., Jeffery J., Ghosh D. (1995). Short-Chain Dehydrogenases/Reductases (SDR). Biochemistry.

[48] Persson B., Kallberg Y., Oppermann U., Jörnvall H. (2003). Coenzyme-Based Functional Assignments of Short-Chain Dehydrogenases/Reductases (SDRs). Chem.-Biol. Interact..

[49] Jörnvall H., von Bahr-Lindström H., Jany K.-D., Ulmer W., Fröschle M. (1984). Extended Superfamily of Short Alcohol-Polyol-Sugar Dehydrogenases: Structural Similarities between Glucose and Ribitol Dehydrogenases. FEBS Lett..

[50] Wierenga R.K., de Maeyer M.C.H., Hol W.G.J. (1985). Interaction of Pyrophosphate Moeieties with α-Helixes in Dinucleotide Binding Proteins. Biochemistry.

[51] Rossman M.G., Liljas A., Branden C.-I., Banaszak L.J., Boyer P.D. (1975). Evolutionary and Structural Relationships among Dehydrogenases. The Enzymes.

[52] Kallberg Y., Oppermann U., Jörnvall H., Persson B. (2002). Short-Chain Dehydrogenases/Reductases (SDRs). Coenzyme-Based Functional Assignments in Completed Genomes. Eur. J. Biochem..

[53] Wu J.T., Wu L.H., Knight J.A. (1986). Stability of NADPH: Effect of Various Factors on the Kinetics of Degradation. Clin. Chem..

[54] Sydenham E.W., Vismer H.F., Walter F.O., Brown N., der Westhuizen V., Rheeder J.P. (1995). Reduction of Patulin in Apple Juice Samples—Influence of Initial Processing. Food Control.

[55] Wickramasinghe S.R., Inglis K.A., Urch J.E., Müller S., van Aalten D.M.F., Fairlamb A.H. (2006). Kinetic, Inhibition and Structural Studies on 3-Oxoacyl-ACP Reductase from *Plasmodium falciparum*, a Key Enzyme in Fatty Acid Biosynthesis. Biochem. J..

[56] Bradford M.M. (1976). A Rapid and Sensitive Method for the Quantitation of Microgram Quantities of Protein Utilizing the Principle of Protein-Dye Binding. Anal. Biochem..

[57] di Costanzo L., Gomez G.A., Christianson D.W. (2007). Crystal Structure of Lactaldehyde Dehydrogenase from *Escherichia coli* and Inferences Regarding Substrate and Cofactor Specificity. J. Mol. Biol..

[58] Fisher M., Kroon J.T., Martindale W., Stuitje A.R., Slabas A.R., Rafferty J.B. (2000). The X-Ray Structure of *Brassica napus* β-Keto Acyl Carrier Protein Reductase and Its Implications for Substrate Binding and Catalysis. Structure.

[59] Price A.C., Zhang Y.M., Rock C.O., White S.W. (2001). Structure of β-Ketoacyl-[Acyl Carrier Protein] Reductase from *Escherichia coli*: Negative Cooperativity and Its Structural Basis. Biochemistry.

[60] Valencia E., Larroy C., Ochoa W.F., Parés X., Fita I., Biosca J.A. (2004). Apo and Holo Structures of an NADP(H)-Dependent Cinnamyl Alcohol Dehydrogenase from *Saccharomyces cerevisiae*. J. Mol. Biol..

[61] Ehrensberger A.H., Wilson D.K. (2004). Structural and Catalytic Diversity in the Two Family 11 Aldo-Keto Reductases. J. Mol. Biol..

