# Supplementary material for: Characterization of Two Dehydrogenases from Gluconobacter oxydans Involved in the Transformation of Patulin to Ascladiol
Source: Toxins (Basel). 2022 Jun 21;14(7):423. doi: 10.3390/toxins14070423 (PMC9323132; doi:10.3390/toxins14070423)
Supplement: Supplementary file 1 [file toxins-14-00423-s001.zip › toxins-1747904-supplementary.pdf]

Supplementary Materials: Characterization of Two Dehydrogenases from *Gluconobacter oxydans* Involved in the Transformation of Patulin to Ascladiol

Edicon T. S. Chan, Yan Zhu, Xiu-Zhen Li, Ting Zhou and Stephen Y. K. Seah

Table S1. Mass Spectrometry Peptide Fingerprinting Results for Fraction 16/17. Putative oxidoreductases are marked with *, NADP(H)-dependent candidates marked with #. The statistical value of -10lgP is calculated by the following formula: $-10*\log_{10}$(P-value), where the P-value is the probability that a false identification has a greater score than the peptide match. PTM stands for post translational modifications.

GENBANK Accession	-10lgP	Coverage (%)	#Peptides	#Unique	PTM	Avg. Mass (Da)	Description
AAW60890.1*	663	100	94	94	Y	50530	Homologous to succinic semialdehyde dehydrogenase
AAW61539.1	476.2	100	29	29	N	22470	Superoxide dismutase [Fe]
AAW61640.1	458.3	78	41	41	Y	57492	Chaperonin GroEL
AAW60688.1	445.2	82	28	28	N	43396	Hypothetical protein GOX0916
AAW61872.1	413.6	67	28	28	N	60796	Aminopeptidase
AAW60648.1	408.6	97	22	22	Y	15555	AtsE protein
AAW60403.1	402.8	99	21	21	Y	11679	Thioredoxin
AAW61452.1*	386.7	57	24	24	Y	52154	Aldehyde dehydrogenase. Predicted NAD(H) dependent Glu177 residue after b2 of Rossman Fold based on sequence alignment with lactaldehyde dehydrogenase of *E. coli* [1].
AAW60303.1*#	372.4	87	20	20	N	25070	Putative oxidoreductase. Predicted NADP(H) dependent Ala35 residue after b2 of Rossman fold based on sequence alignment with *E. coli* and *Brassica napus* β-keto acyl carrier protein [2]. Arg13 corresponds to Arg residues in *E. coli* and *B. napus* β-keto acyl carrier protein that forms a salt bridge with 2′-adenosine ribose phosphate of NADP(H) [3].
AAW60280.1*	362.7	63	17	17	N	39242	Putative oxidoreductase. FMN dependent
AAW61580.1	359.9	99	12	12	N	8834	Hypothetical protein GOX1841
AAW60778.1	357.8	54	12	12	N	15542	Hypothetical protein GOX1007
AAW60503.1	332.1	50	13	13	Y	23289	Hypothetical protein GOX0726
AAW61665.1	304.9	99	14	14	N	15176	Nucleoside diphosphate kinase
AAW61175.1	276.9	99	9	9	N	7952	Hypothetical protein GOX1424
AAW61728.1	181.7	41	4	4	N	15093	Osmotically inducible protein C
AAW61437.1	158.2	16	2	2	N	19384	Hypothetical protein GOX1697
AAW59880.1	150.5	50	3	3	N	9939	DNA-binding protein HU
AAW61552.1	124.6	34	3	3	N	20822	Ribosome Recycling Factor (RRF)
AAW60165.1	77.68	13	2	2	N	42981	Protein Translation Elongation Factor Tu
AAW61512.1	67.43	10	2	2	N	33037	Putative LacX protein

Table S2. Mass Spectrometry Peptide Fingerprinting Results for Fraction 20. Putative oxidoreductases are marked with *, NADP(H)-dependent candidates marked with #. The statistical value of -10lgP is calculated by the following formula: -10*log10(P-value), where the P-value is the probability that a false identification has a greater score than the peptide match. PTM stands for post translational modifications.

Accession	-10lgP	Coverage (%)	#Peptides	#Unique	PTM	Avg. Mass (Da)	Description
AAW61770.1	528.3	70	59	59	N	77324	Metalloprotease
AAW60096.1*	457	62	22	22	N	36462	NAD-dependent alcohol dehydrogenase. Predicted NAD(H) dependent<br>Asp200 residue after b2 of Rossman fold based on sequence alignment with cinnamyl alcohol dehydrogenase of *Saccharomyces cerevisiae* [4].
AAW61640.1	424.7	74	36	36	N	57492	Chaperonin GroEL
AAW60639.1*	417.9	73	22	22	N	27802	Xylitol Dehydrogenase. Predicted NAD(H) dependent<br>Asp28 residue after b2 of Rossman fold based on sequence alignment with *E. coli* and *Brassica napus* β-keto acyl carrier protein [2].
AAW61941.1	403	69	30	30	N	38297	5-Methyltetrahydropteroyltriglutamate--homocysteine S-methyltransferase
AAW61637.1*#	384.8	84	20	20	N	26560	Putative oxidoreductase. Predicted NADP(H) dependent<br>Ala 39 residue after b2 of Rossman fold based on sequence alignment with *E. coli* and *Brassica napus* β-keto acyl carrier protein [2].
AAW61231.1	347.8	53	19	19	N	44033	UDP-galactopyranose mutase
AAW60997.1	324.6	64	13	13	N	36904	Ornithine carbamoyltransferase
AAW61984.1	321.9	45	20	20	N	51412	Pyruvate kinase
AAW60097.1*#	318.2	51	14	14	N	35366	Probable alcohol dehydrogenase-like oxidoreductase protein. Predicted NADP(H)-dependent<br>Thr 198 residue after b2 of Rossman fold based on sequence alignment with cinnamyl alcohol dehydrogenase of *Saccharomyces cerevisiae* [4].
AAW60211.1	314.1	40	14	14	N	55380	Phosphoribosylaminoimidazolecarboxamide formyltransferase/IMP cyclohydrolase
AAW60484.1	297.4	77	12	12	N	19722	Starvation-inducible DNA-binding protein
AAW59748.1*	282.5	51	13	13	N	39361	NAD(P)H-dependent 2-cyclohexen-1-one reductase (plasmid). Homologous to old yellow enzyme (Flavin-dependent)
AAW60636.1	261.5	48	9	9	N	21198	Acyl carrier protein phosphodiesterase
AAW60821.1	253.2	48	10	10	N	21136	dTDP-4-dehydrorhamnose 3,5-epimerase
AAW60692.1	246.5	37	7	7	N	35724	Phosphate-binding periplasmic protein precursor
AAW61054.1	236.6	26	7	7	N	41591	Glutamyl-tRNA(Gln) amidotransferase subunit A
AAW61447.1	228.8	66	11	11	N	26315	6-Phosphogluconolactonase
AAW60170.1	227.6	47	9	9	N	12696	LSU ribosomal protein L12P (L7/L12)
AAW60919.1	224.8	90	9	9	N	12919	Hypothetical protein GOX1152
AAW61356.1*#	222.3	32	8	8	N	37213	Putative oxidoreductase. Homologous to Aldo-keto reductase family 11 enzymes (AKR11). Predicted NADP(H) dependent<br>Lys217 corresponds to Lys214 of *Bacillus subtilis* AKR11a and AKR11b, Arg 230 correspond to Arg 227 of AKR11b. Positive

							charged residues in AKR11 interact with 2′-phsphate of NADPH [5].
AAW60213.1	214.1	37	7	7	N	21297	KDPG aldolase
AAW61870.1	209.7	9	6	6	N	79735	Peptidyl-dipeptidase DCP
AAW61417.1	208.2	29	10	10	N	32331	Peptidyl-prolyl cis-trans isomerase precursor
AAW60444.1	200.7	71	5	5	N	7249	Copper resistance protein CopZ
AAW61888.1	188.5	48	4	4	N	13347	Hypothetical protein GOX2152
AAW60329.1	184.9	42	6	6	N	28727	Putative hydrolase
AAW61230.1	184.6	32	5	5	N	35810	UDP-glucose 4-epimerase
AAW60648.1	182.9	65	5	5	N	15555	AtsE protein
AAW60643.1	175.8	20	5	5	N	26531	Electron transfer flavoprotein beta-subunit
AAW60779.1	173.7	34	5	5	N	22204	Hypothetical protein GOX1008
AAW60601.1	173.7	33	5	5	N	26010	Hypothetical protein GOX0827
AAW61059.1	171.1	23	5	5	N	44254	Serine--pyruvate aminotransferase
AAW61369.1	169	19	5	5	N	21434	Protease
AAW61084.1	167.1	23	4	4	N	20548	Alkyl hydroperoxide reductase subunit C
AAW61686.1	165.9	18	6	6	N	58926	GMP synthase [glutamine-hydrolyzing]
AAW61438.1	165.4	17	5	5	N	46232	Aminopeptidase
AAW61342.1	161.7	20	6	6	N	36855	Cysteine synthase A (O-acetylserine sulfhydrylase A) protein
AAW60200.1*	158	29	5	5	N	33146	Putative 2-hydroxyacid dehydrogenase. Homologous to hydroxy(phenyl)pyruvate reductases that transform ketones to alcohols.
AAW60850.1	155.9	7	4	4	N	60734	Pyruvate decarboxylase
AAW61211.1*#	155.8	33	7	7	Y	40013	Putative oxidoreductase. Homologous to Aldo-keto reductase family 11 enzymes (AKR11). Predicted NADP(H) dependent Lys222 corresponds to Lys214 of *Bacillus subtilis* AKR11a and AKR11b, Arg 235 correspond to Arg 227 of AKR11b. Positive charged residues in AKR11 interact with 2′-phsphate of NADPH [5]
AAW61724.1	153.7	29	3	3	N	22656	Pyridoxamine 5'-phosphate oxidase
AAW60661.1	151.9	37	6	6	N	17280	Peptidyl-prolyl cis-trans isomerase
AAW60608.1*	137.7	11	3	3	N	30076	Putative oxidoreductase. Homology to nitroreductases
AAW61563.1	129.7	29	4	4	N	14646	Lactoylglutathione lyase
AAW60233.1	128.7	22	4	4	N	41393	Branched-chain amino acid aminotransferase
AAW61209.1	128.4	21	3	3	N	23976	Glutaredoxin 2
AAW61467.1	124.6	21	4	4	Y	42624	Phosphoribosylamine-glycine ligase
AAW61279.1	108.8	17	2	2	N	24586	GTP cyclohydrolase I
AAW60000.1	107.8	9	2	2	N	27165	Lactam utilization protein LamB
AAW60807.1	103.9	24	4	4	N	25515	Putative phosphatase
AAW61171.1	86.18	9	2	2	N	19292	Putative peroxiredoxin
AAW61037.1	81.51	2	1	1	N	49006	Organophopsphate acid anhydrase
AAW61195.1	80.82	5	2	2	N	41733	Phosphoserine aminotransferase
AAW61741.1	80.69	16	2	2	N	30226	Putative thiol:disulfide interchange protein
AAW59808.1	71.65	11	2	2	N	22800	Thiamin-phosphate pyrophosphorylase
AAW61971.1	67.77	26	2	2	N	19082	Protein translocase subunit SecB
AAW61384.1	62.03	3	2	2	N	50637	Fumarate hydratase
AAW60753.1	54.82	11	1	1	N	17089	6,7-Dimethyl-8-ribityllumazine synthase
AAW60872.1	54.46	3	1	1	N	42244	Transaminase
AAW61130.1	51.3	7	1	1	N	16620	Hypothetical protein GOX1378

AAW60493.1*#	45.27	4	1	1	N	26960	Putative oxidoreductase. Predicted NADP(H)-dependent His39 residue after b2 of Rossman fold based on sequence alignment with *E. coli* and *Brassica napus* β-keto acyl carrier protein [2].
AAW60822.1	39.39	8	1	1	N	11354	Glucose-1-phosphate thymidylyltransferase, C-terminus
AAW60527.1	38.2	5	1	1	N	21485	Hypothetical protein GOX0750
AAW60219.1	36.91	22	1	1	N	15131	Biotin carboxyl carrier protein of acetyl-CoA carboxylase
AAW60115.1	36.59	3	1	1	N	51236	Carboxy-terminal protease
AAW61740.1	32.54	4	1	1	N	23206	Putative thiol:disulfide interchange protein
AAW60631.1	31.5	1	1	1	N	67711	Chaperone protein DnaK +H65:H76
AAW60818.1	30.8	8	1	1	N	15925	Hypothetical protein GOX1048

.References

1. di Costanzo, L.; Gomez, G.A.; Christianson, D.W. Crystal Structure of Lactaldehyde Dehydrogenase from *Escherichia coli* and Inferences Regarding Substrate and Cofactor Specificity. *J Mol Biol.* **2007**, *366*, 481–493.
2. Fisher, M.; Kroon, J.T.; Martindale, W.; Stuitje, A.R.; Slabas, A.R.; Rafferty, J.B. The X-Ray Structure of *Brassica napus* β-Keto Acyl Carrier Protein Reductase and Its Implications for Substrate Binding and Catalysis. *Structure* **2000**, *8*, 339–347, doi:10.1016/S0969-2126(00)00115-5.
3. Price, A.C.; Zhang, Y.M.; Rock, C.O.; White, S.W. Structure of β-Ketoacyl-[Acyl Carrier Protein] Reductase from *Escherichia coli*: Negative Cooperativity and Its Structural Basis. *Biochemistry* **2001**, *40*, 12772–12781, doi:10.1021/bi010737g.
4. Valencia, E.; Larroy, C.; Ochoa, W.F.; Parés, X.; Fita, I.; Biosca, J.A. Apo and Holo Structures of an NADP(H)-Dependent Cinnamyl Alcohol Dehydrogenase from *Saccharomyces cerevisiae*. *Journal of Molecular Biology* **2004**, *341*, 1049–1062, doi:10.1016/j.jmb.2004.06.037.
5. Ehrensberger, A.H.; Wilson, D.K. Structural and Catalytic Diversity in the Two Family 11 Aldo-Keto Reductases. *Journal of Molecular Biology* **2004**, *337*, 661–673, doi:10.1016/j.jmb.2004.01.059.
